# Effect of Fiber Hybridization, Strain Rate and *w/c* Ratio on the Impact Behavior of Hybrid FRC

**DOI:** 10.3390/ma12172780

**Published:** 2019-08-29

**Authors:** Lei Wang, Hua Zhang, Lingyu Bai, Heang Hong, Jie Tao, Maxwell Addae

**Affiliations:** College of Civil and Transportation Engineering, Hohai University, Nanjing 210098, China

**Keywords:** hybrid FRC, SHPB, impact behavior, damage dynamic constitutive model, microstructure characteristics

## Abstract

Concrete in practical applications has to inevitably suffer various impact loads. Recent research indicates that the hybrid fiber reinforced concrete (FRC) has better dynamic mechanical properties compared to the mono FRC under impact loading. Based on macro-experimentation and micro-observation, the impact behavior of the hybrid basalt-macro synthetic polypropylene FRC (BSFRC) was investigated by using ∅74 mm SHPB, SEM, and EDS. The effects of fiber hybridization, strain rate, and *w/c* ratio were analyzed simultaneously. The results show that the dynamic mechanical properties of BSFRC are strain-rate sensitive. Both basalt and macro synthetic polypropylene fibers (BF, SF) have a strengthening and toughening effect on concrete. Their hybridization has a similar enhancement effect but the impact toughness of concrete is further improved and the best hybrid ratio is 0.05%(BF)–0.25%(SF). BSFRC with higher *w/c* ratio has a higher strain rate effect while the fiber hybridization effect is weakened. Besides, the proposed constitutive model can well describe the impact behavior of BSFRC. The hydration of cement in the interface transition zones is lower with more Calcium Silicate Hydrate and less $\mathrm{Ca}{\left(\mathrm{OH}\right)}_{2}$ than that in the common mortar. However, the addition of BF and SF contributes to the hydration of cement and improves the performance of concrete eventually.

## 1. Introduction

Concrete is a widely applied engineering material and measures to improve the performance of concrete are desperately needed. Fiber reinforced concrete (FRC) has been proven to have better performance than plain concrete (PC), especially in restraining microcracks propagation [1]. Based on elastic modulus, fibers can be classified as rigid fibers and flexible fibers. While mixed together, the hybrid FRC shows a better strengthening and toughening effect than the mono FRC [2,3]. Among the various fibers, basalt fiber (BF) and macro synthetic polypropylene fiber (SF) are receiving more and more attention. Studies have proven that concrete reinforced with BF or SF has better mechanical performance and durability [4,5]. SF (diameter > 100 μm [6]), which is a great substitute for steel fiber, is made of modified polypropylene. With the advantages of lighter weight and higher corrosion resistance [7], SF can prevent drying shrinkage cracking of concrete [4] and is expected to improve the durability and lifetime of concrete structures [8]. Besides, SF is easy to disperse with less agglomeration in concrete compared to polypropylene fiber [9]. BF is extracted from volcanic basalt rock and is a kind of environmentally friendly material [5], possessing high elastic modulus, tensile strength, and excellent resistance to alkaline and high temperature [10]. When BF is added, not only will the tensile and flexural strength of concrete be improved remarkably [11], but also the permeability and dry shrinkage will be restricted [12,13]. So together with its low cost, BF has high potential in concrete application [14,15].

There are few studies on the hybrid basalt-macro synthetic polypropylene FRC (BSFRC) at present, especially on its dynamic mechanical properties. However, other types of HFRC have also been studied. Yu et al. [16] studied the impact resistance of ultra-high performance concrete reinforced with long and short steel fibers (UHPHFRC). The results showed that the hybrid FRC had higher workability and the flexural and compressive strength were improved by 82% and 43% with long steel fibers (1.5%) and short steel fibers (0.5%). Ali et al. [17] investigated the behavior of an innovative engineered cementitious composite (ECC) reinforced with shape memory alloy (SMA) and PVA fibers. The results showed that hybrid fiber ECC became more brittle and its impact resistance was higher than plain ECC and the ECC with fiber hybridization of 2%(PVA)–1%(SMA) achieved the highest impact resistance. However, this research did not analyze the effect of different fiber hybridization. From the perspective of the fibers’ chemical and physical properties, Pakravan et al. [18] investigated the impact of fiber hybridization on account of the recent studies on HFRC.

Besides, most of the research is basically carried out at the macro scale now. However, some limitations exist when explaining the impact of fiber and strain rate upon the dynamic behavior of concrete material. Scanning electron microscopy (SEM) is an advanced processing method for observing sample surfaces and is widely applied for analyzing the microstructure characteristics of materials [19]. It is generally known that the macroscopic mechanical properties of concrete are deeply affected by its the microstructure. By combining experimental procedures and SEM, we can further understand the mechanism of concrete failure. Zhang et al. [20] utilized SEM to analyze the interface between BF and mortar and found that concrete of higher strength had better bonding interfaces. Fiber inside concrete could not improve the interface performance but it had the ability to reduce the micro defects of concrete, such as microcracks and pores. Behfarnia [21] studied the effect of polypropylene fiber (PF) upon the microstructural appearance of concrete. The SEM images showed that PF had both positive and negative effects on mechanical and durability-related properties of concrete. It can be deduced that interfaces between mortar and fiber, mortar, and aggregates are important factors affecting concrete failure. Therefore, research on the microstructural characteristics of specimens is significant to understand the failure mechanism of BSFRC under impact loading.

**Figure 1:**
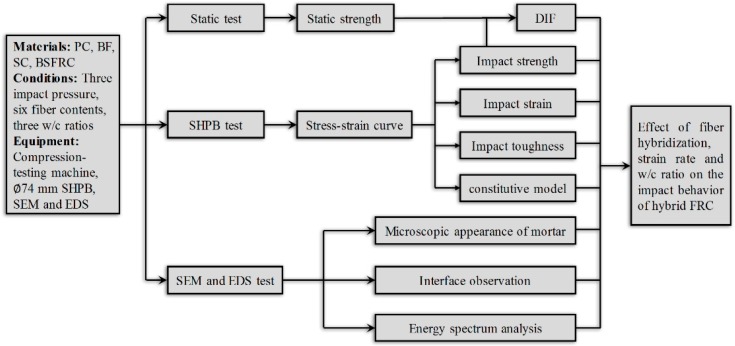
The methodological framework flow chart.

This paper is devoted to analyzing the impact behavior and failure mechanism of BSFRC from both macro and micro perspectives. The effect of fiber hybridization, strain rate, and *w/c* ratio was investigated simultaneously with a split Hopkinson pressure bar (SHPB) device. Then, a proposed model for BSFRC was used to describe the mechanical behavior of BSFRC considering damage inside concrete. Finally, the microstructure characteristics of BSFRC were observed and analyzed according to the obtained microscopic appearance and element contents in the interface transition zones (ITZ). The methodological framework chart of this study is shown as Figure 1.

## 2. Experiment Framework

### 2.1. Materials and Specimens Preparation

**Figure 2:**
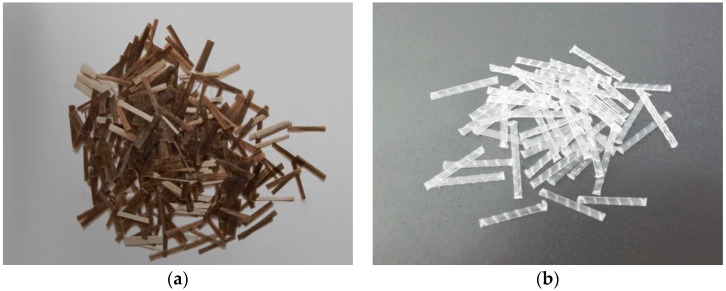
Basalt fiber and macro synthetic fiber: (**a**) basalt fiber (BF); (**b**) macro synthetic polypropylene fiber (SF).

The *w/c* ratios of 0.68, 0.54 and 0.44 were considered. Six groups of fiber hybrid content were designed which were PC (0%), BC (0.1% of BF), SC (0.5% of SF), BSC1 (0.05% of BF + 0.25% of SF), BSC2 (0.05% of BF + 0.5% of SF), BSC3 (0.1% of BF + 0.25% of SF). The strain rate was controlled by impact pressures in SHPB test which were 0.3, 0.4, and 0.5 MPa, respectively. The river sands were used as fine aggregates with the maximum particle size of 2.5 mm. The detritus was used as coarse aggregates with a particle size of 5–10 mm. The 42.5 ordinary Portland cement was used according to GB 175-2009 [22]. The mixture proportion for concrete is listed in Table 1. The properties of BF and SF, as shown in Figure 2, are given in Table 2. No other materials were used. Table 3 tabulates all the test conditions.

**Figure 3:**
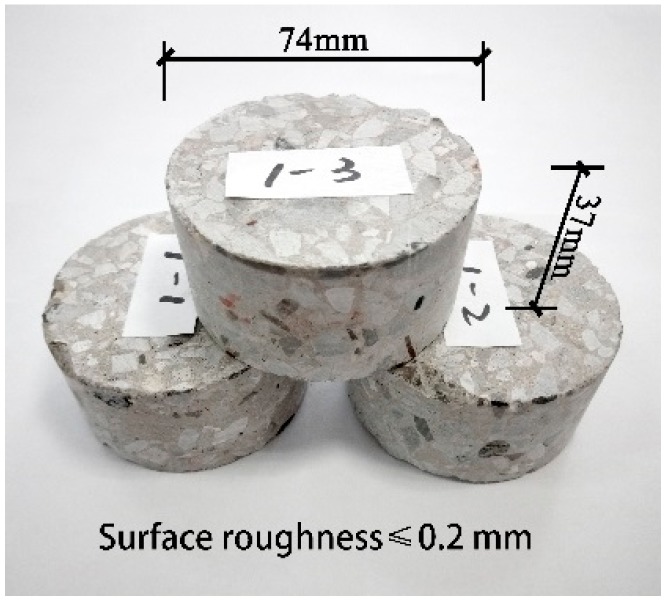
Three identical SHPB specimens of each condition.

The size of cylinder specimens was an important factor in SHPB test. Li et al. [23] considered that the cylinder aspect ratio (H/D) was about 0.5 as the inertial and terminal effects were restrained greatly. Herein, the height and diameter were 37 mm and 74 mm. The static specimens were 150 mm cubes. In this study, a hand-propelled mixer is used. The mixing procedure is that detritus and fibers were mixed for about 30 s firstly. Then, cement and sand were successively added and mixed for 90 s. Finally, water was added and the mixing continued for 3 min. Overall, the mixing time of FRC is 1 min longer than that of plain concrete to make fibers disperse evenly in concrete. The concrete was poured in 150 mm cubic molds, demolded two days later, and then cured in water at 20 ± 2 °C to 28 days. Part of the cubes were machined into ∅74 mm × 37 mm cylinders after being cored, sliced, and polished for SHPB test, as shown in Figure 3. The static test was performed with a compression-testing machine at the speed of 3 × 10^−5^ MPa/s according to GB/T 50081-2016 [24].

**Figure 4:**
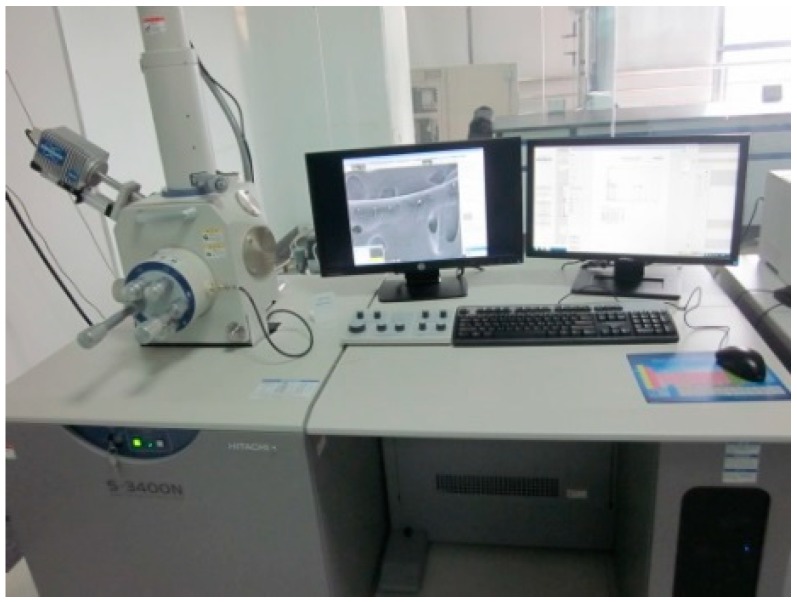
The JSM-6360LV digital scanning electron microscope.

**Figure 5:**
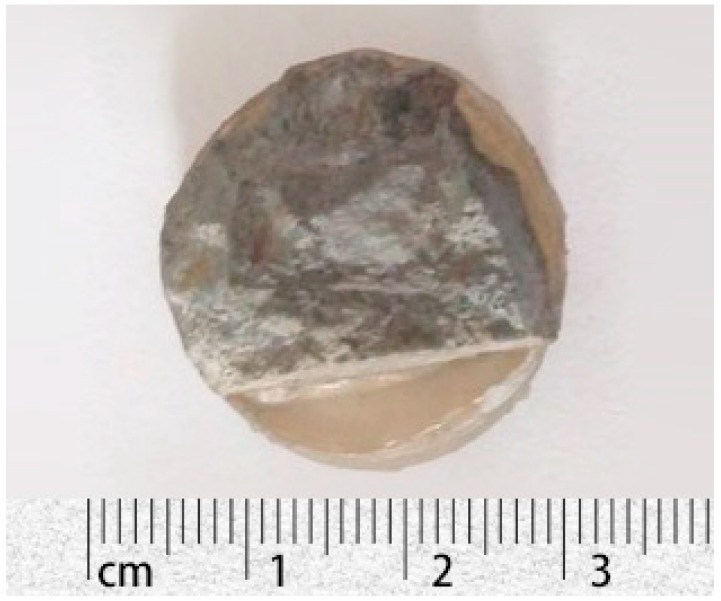
The SEM specimen.

The SEM test was conducted to study the microscopic appearance of BSFRC with a JSM-6360LV digital scanning electron microscope (JEOL Ltd., Tokyo, Japan), as shown in Figure 4. The SEM specimens should be as small as possible but must contain fibers, detritus, and interfaces. The size of SEM specimens was about 25 mm in this study. After the SHPB test, the selected specimens were inlaid with epoxy resin to make a flat surface for fixing. Then, they were polished and dried in a vacuum drying oven. Lastly, they were sprayed with gold conductive coating for better imaging quality. The obtained SEM specimen is shown in Figure 5. The energy dispersive spectrometer (EDS) was used to study the element types and contents in the ITZ of BSFRC. This technique is based on the energy analysis of X-Rays from excited elements within the volume of the electron–solid interaction volume as each element has a unique atomic structure allowing unique set of peaks on its X-ray emission spectrum [25,26]. The number and energy of the X-rays emitted from a specimen can be measured by EDS. As the energies of the X-rays are characteristic of the difference in energy between the two shells and of the atomic structure of the emitting element, EDS allows the elemental composition of the specimen to be measured.

### 2.2. Basic Principle of SHPB Test

B. Hopkinson [27] in 1914 proposed a Hopkinson pressure bar (HPB) apparatus to measure the impact elastic pulses of metallic materials and Kolsky [28] subsequently developed it into SHPB in 1949. Compared to other dynamic test methods (e.g., hydraulic, falling hammer, light gas gun), the SHPB test not only has a relatively simple device and a wide range of strain rate, but also it is easy to control the waveform and it can be carried out in various forms of experiments.

**Figure 6:**
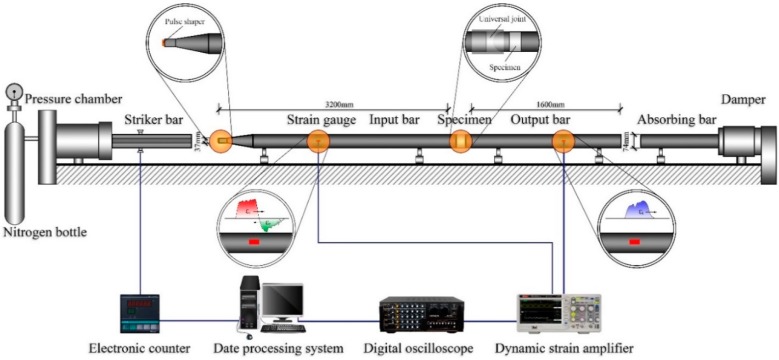
Schematic illustration of SHPB apparatus.

**Figure 7:**
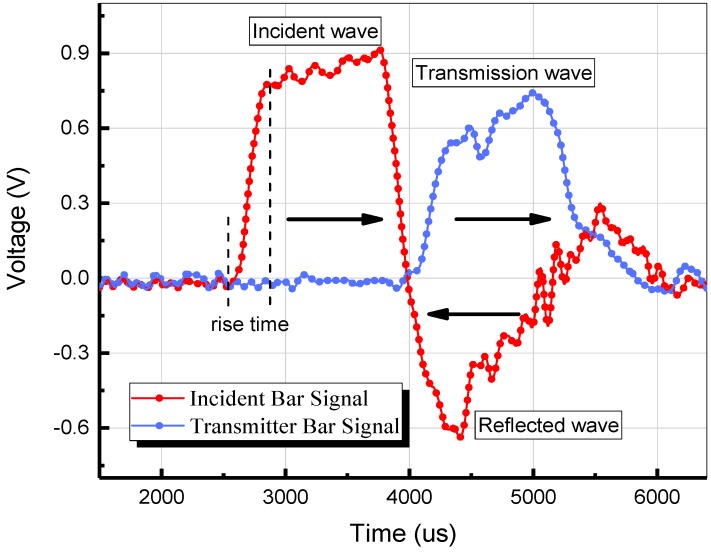
Typical detected pulses.

The SHPB apparatus used in this study is illustrated in Figure 6. Strain gauges are used to detect the incident, reflected, and transmitted waves ($\varepsilon_{i}$, $\varepsilon_{r}$, $\varepsilon_{t}$). The pulse shapers were utilized to increase the rise time and stress uniformity [29]. The specimen was placed between the bars with a universal joint for better contact surfaces. The typical detected waves are shown in Figure 7. Based on the 1D wave theory and the uniformity assumption ($\varepsilon_{i}+\varepsilon_{r}=\varepsilon_{t}$), the strain (ε), stress (σ) and strain rate ($\dot{\varepsilon }$) can be obtained by the Equations (1)–(3) [30]:

(1) $$\sigma =E\frac{A}{A_{s}}\varepsilon_{t}$$

(2) $$\varepsilon =-\frac{2c_{0}}{l_{0}}\int_{0}^{t}\varepsilon_{r}dt$$

(3) $$\dot{\varepsilon }=-\frac{2c_{0}}{l_{0}}\varepsilon_{r}$$

where *E* and *A* are the elastic constant and sectional area of the input and output bars. $l_{0}$ and $A_{s}$ are the length and sectional area of the specimen. $c_{0}$ is the wave velocity.

## 3. Discussions

### 3.1. Static Test

**Figure 8:**
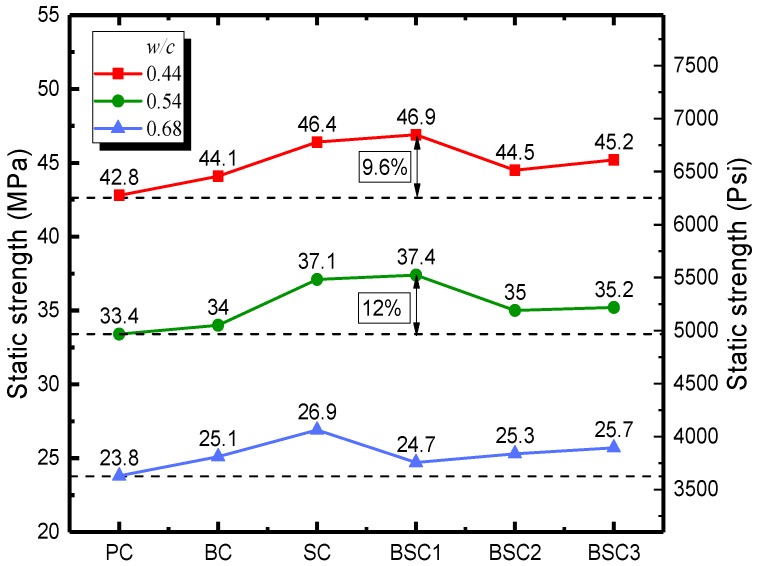
The 28-day compressive strength of BSFRC.

**Figure 9:**
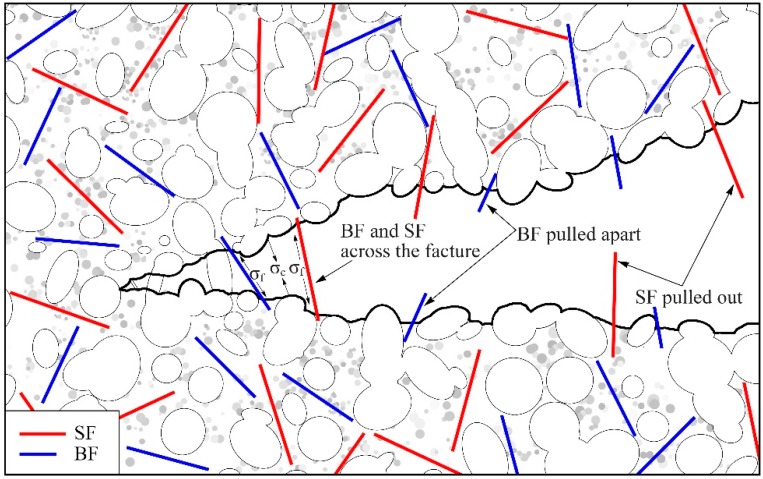
BF and SF distribution in crack area.

The static strength of concrete is an important factor in designing concrete structures and is the basis for analyzing the dynamic properties of concrete. Figure 8 plots the static strength of concrete with various *w/c* ratios and fiber hybrid ratios. It shows that the strength of all types of concrete reinforced with fibers is higher than that of fiberless concrete. Giner et al. [31] considered that the introduction of steel and carbon fiber to concrete would increase the porosity and air content leading to the decrease in strength. Instead, the results in this study show that proper content of fibers can increase the strength of concrete. It is because that the fibers across the cracks can bridge the upper and lower concrete, as illustrated in Figure 9. The tension in crack area is counteracted and the propagation of cracks are restrained by BF and SF. The static strength is improved accordingly. For *w/c* ratios of 0.44 and 0.54, the carrying capacity of BSC1 (0.5BC + 0.5SC) is higher than that of BC and SC which are increased by 9.6% and 12%, respectively. It indicates that the hybrid fiber has greater enhancement effect than mono fiber. However, the excessive fiber hybrid content (BSC2 and BSC3) produces inhibitory effects and the strength of concrete decreases. This is because too much fiber results in poor dispersibility and conglomeration. More defects of pores and weak interfaces are produced, leading to the weakness of enhancement effect of fibers. For *w/c* ratio of 0.68, bearing capacity of BSC1 is lower than that of BC and SC. Whereas, the strength of concrete continues to increase when the content of BF (BSC2) or SF (BSC3) is increased. This is probably because concrete with higher *w/c* ratio demands a higher fiber hybrid content. Concrete with low *w/c* ratio and high fiber content must use superplasticizers. Overall, the optimal hybrid ratio is 0.05% (BF)–0.25% (SF) for *w/c* ratios of 0.44 and 0.54, but higher fiber hybrid content is needed for a *w/c* ratio of 0.68.

### 3.2. Impact Test

**Figure 10:**
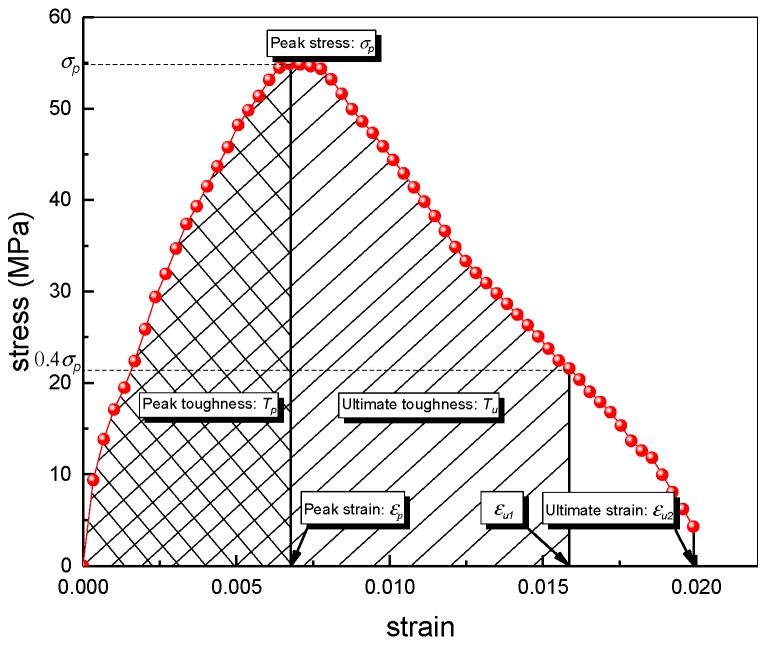
Dynamic characteristic parameters in the stress–strain curve of the SHPB test.

**Figure 11:**
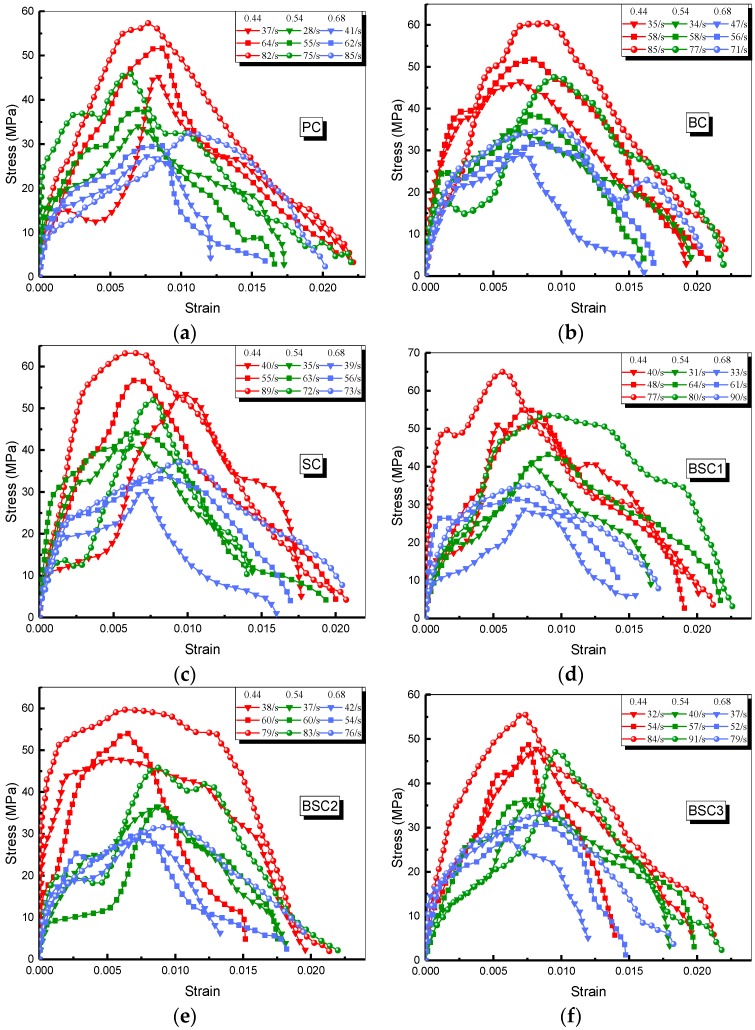
Impact stress–strain curves in the SHPB test: (**a**) PC; (**b**) BC; (**c**) SC; (**d**) BSC1; (**e**) BSC2; (**f**) BSC3.

Here, the impact of fiber hybridization, strain rate and *w/c* ratio on the dynamic compressive response of BSFRC subjected to dynamic compressive loading is investigated. The dynamic characteristic parameters of SHPB test are defined in Figure 10. Figure 11 and Table 4 present all SHPB test results.

#### 3.2.1. Strain Rate Effect

**Figure 12:**
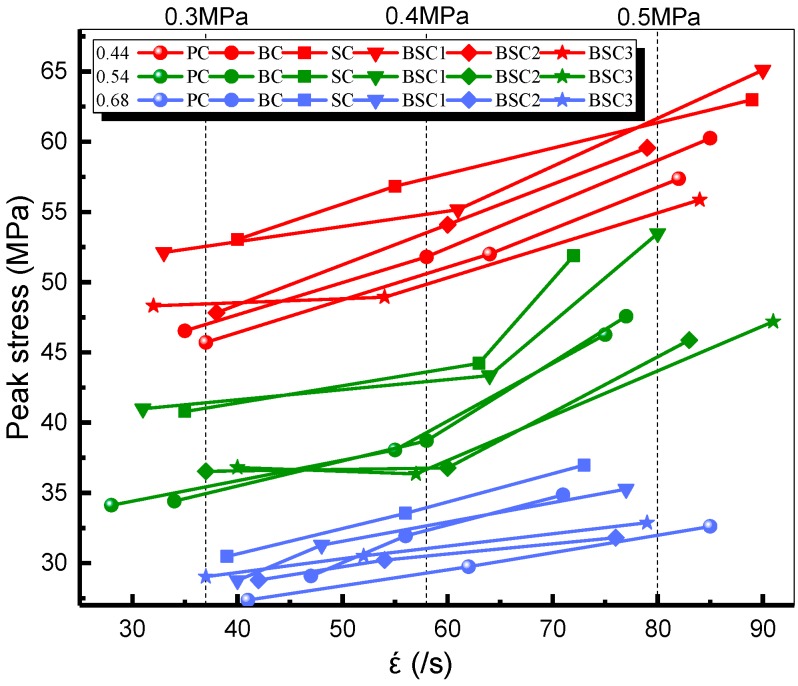
Strain rate effect on the peak stress of BSFRC.

Impact strength ($\sigma_{p}$) represents the impact resistance of BSFRC material and it is the maximum value in stress–strain curves. Figure 12 indicates that the dynamic strength of BSFRC was improved with increasing strain rate under different conditions. It is known that higher impact pressures bring about higher impact loading and shorter impact time acting on specimens. New cracks inside have no time to extend fully and the lateral deformation is restrained due to the Poisson effect. The surrounding part generates lateral confining forces to the central part of BSFRC [32,33] which cannot be neglected. Thus, the stress is improved to absorb the input energy and the dynamic strength of BSFRC is improved eventually. It is found that curves of *w/c* ratios of 0.68 and 0.44 are approximated as straight lines. However, curves of *w/c* ratio of 0.54 have obvious inflection points. Some scholars call it as critical strain rate (${\dot{\varepsilon }}_{c}$) [34]. The impact strength of BSFRC increases slowly when strain rate is lower than ${\dot{\varepsilon }}_{c}$ but increases rapidly when strain rate is greater than ${\dot{\varepsilon }}_{c}$. The strain-rate sensitivity of BSFRC material changes to higher with strain rate.

**Figure 13:**
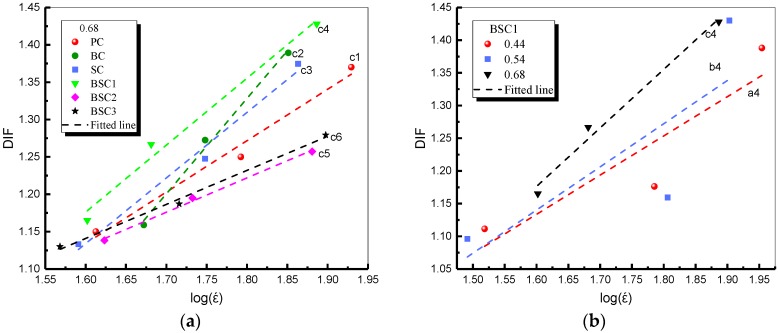
Strain rate effect on DIF of BSFRC: (**a**) *w/c* ratio: 0.68; (**b**) BSC1.

Dynamic increase factor (DIF) shows the change degree of impact strength with strain rate and it is defined as $DIF=f_{cd}/f_{cs}$, where $f_{cd}$ is impact strength and $f_{cs}$ is static strength. Scholars have proposed some expressions to describe the DIF and strain rate relationship, as given in Equations (4)–(8) [32,35,36,37,38,39]. After comparison, the linear expression Equation (6) was finally chosen in this study and the results are given in Figure 13 and Table 5. Figure 13a shows that BSC1 has the highest values. BC and SC are similar but higher than PC. BSC2 and BSC3 are similar but lower than PC. It indicates that the two fibers can increase the impact resistance of concrete and their hybridization has better enhancement. Whereas, excessive fiber hybrid content will play an inhibitory role. From Figure 13b, it is found that the values of DIF increase and the fitted line becomes steeper as *w/c* ratio increases. It means that BSFRC with higher *w/c* ratio has a higher strain rate effect. R^2^ is the determination coefficient which can qualify the fitting results. Here, R^2^ close to 1 means that DIF and $\mathrm{lg}\dot{\varepsilon }$ have an obvious linear relationship.

(4) $$\mathrm{DIF}=\{\begin{array}{ll} {\left(\dot{\varepsilon }/{\dot{\varepsilon }}_{0}\right)}^{0.014} & \dot{\varepsilon }\leq 30/s\\ 0.012{\left(\dot{\varepsilon }/{\dot{\varepsilon }}_{0}\right)}^{1/3} & \dot{\varepsilon }>30/s \end{array}$$

(5) $$\mathrm{DIF}={\left(\dot{\varepsilon }/{\dot{\varepsilon }}_{s}\right)}^{0.006{\left[\log\left(\dot{\varepsilon }/{\dot{\varepsilon }}_{s}\right)\right]}^{1.05}}$$

(6) $$\mathrm{DIF}=\{\begin{array}{ll} 0.00965\cdot lg\dot{\varepsilon }+1.058\geq 1.0 & \dot{\varepsilon }\leq 63.1/s\\ 0.758\cdot lg\dot{\varepsilon }-0.289\leq 2.5 & \dot{\varepsilon }\geq 63.1/s \end{array}$$

(7) $$\mathrm{DIF}=\{\begin{array}{ll} 0.03438\cdot \left(3+lg\dot{\varepsilon }\right)+1 & \dot{\varepsilon }\leq 100/s\\ 1.729\cdot {\left(\mathrm{lg}\dot{\varepsilon }\right)}^{2}-7.1372\cdot lg\dot{\varepsilon }+8.5303 & \dot{\varepsilon }>250/s \end{array}$$

(8) $$\mathrm{DIF}=\left(3.54\cdot \dot{\varepsilon }+430.6\right)/\left(\dot{\varepsilon }+447.3\right)$$

**Figure 14:**
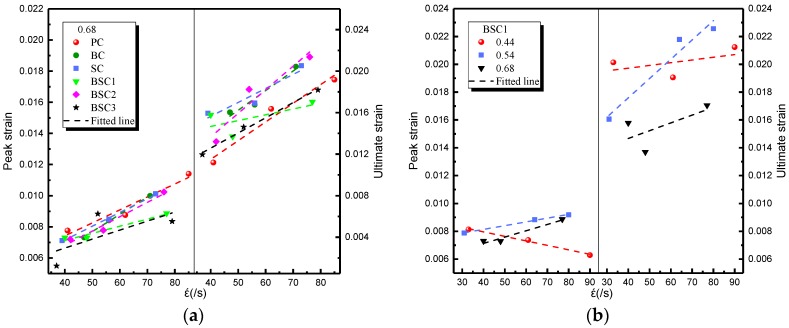
Strain rate effect on impact strain of BSFRC: (**a**) *w/c* ratio: 0.68; (**b**) BSC1.

The impact strain indicates the deformation capacity of BSFRC under impact loading. The relationship between strain rate and impact strain is presented in Figure 14. It shows that the impact strain of BSFRC is improved by strain rate. This is inconsistent with the strain-rate hardening effect [40], namely, the strain of strain-rate sensitive material will be restrained as the stress increases. This is because BSFRC is heterogeneous and the strain softening effect [41] appears due to the microcracks and pores inside BSFRC. In fact, both strain-rate hardening effect and strain softening effect play a significant role in the failure of BSFRC but the former is dominant. This is consistent with the conclusions obtained by Zhang et al. [42]. In terms of the effect of *w/c* ratio, it shows from Figure 14b that the impact strain of *w/c* ratio of 0.54 has the highest value but that of 0.44 has a downward trend. As is known, the brittleness of concrete increases when the *w/c* ratio decreases. It means that the strain-rate hardening effect is dominant as the *w/c* ratio of BSFRC is low.

**Figure 15:**
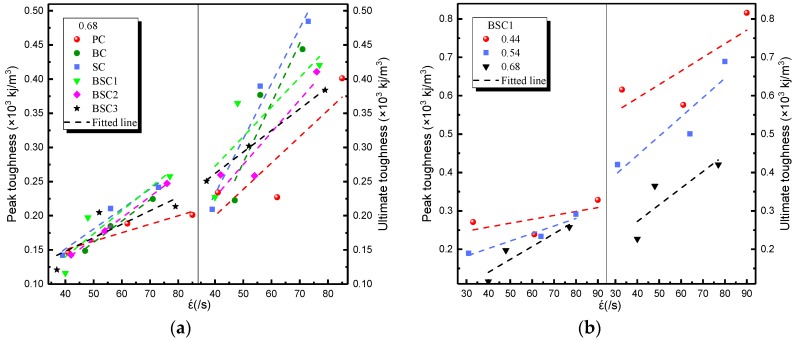
Strain rate effect on impact toughness of BSFRC: (**a**) *w/c* ratio: 0.68; (**b**) BSC1.

The impact toughness reflects the energy absorption capacity of BSFRC subjected to external loading. Herein, we use the energy method to analyze the impact toughness of BSFRC ($T_{p}=\int_{0}^{\varepsilon_{p}}\sigma d\varepsilon$, $T_{u}=\int_{0}^{\varepsilon_{u1}}\sigma d\varepsilon$, as presented in Figure 10. Figure 15 gives the relationship between strain rate and the peak and ultimate toughness of concrete. It shows that the impact toughness of BSFRC specimens is improved by strain rate. On the one hand, the impact toughness of BSFRC is determined by the dynamic strength and impact strain. Both of them are enhanced by strain rate, leading to higher impact toughness of BSFRC eventually. On the other hand, the propagation of cracks accelerates directly the failure of concrete. The appearance of new microcracks consumes much more external energy than cracks expansion [20]. With increasing strain rate, more cracks appear to consume input energy and hence create the stress relaxation areas. The macroscopic manifestation is that the specimen appears to sustain more severe damage. Figure 15b shows that the peak and ultimate toughness are weakened as the *w/c* ratio increases. However, the fitted line becomes steeper as *w/c* ratio increases. It also indicates that BSFRC with higher *w/c* ratio has a higher strain rate effect.

#### 3.2.2. Fiber Hybridization Effect

**Figure 16:**
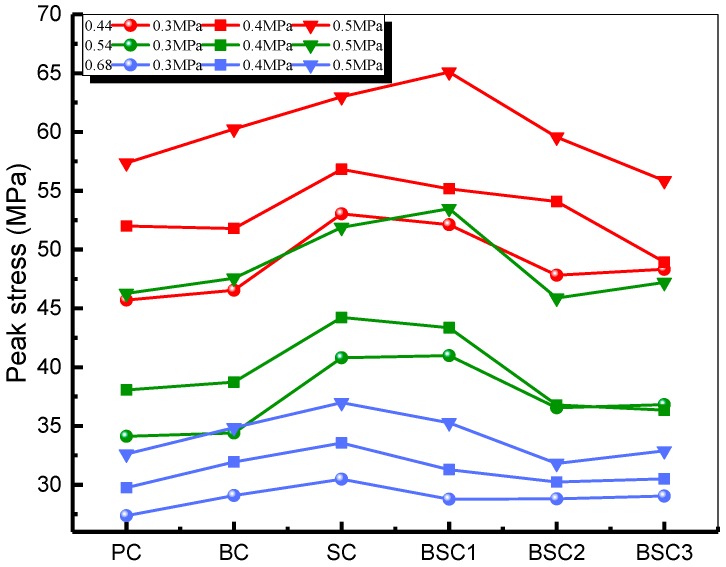
Fiber hybridization effect on impact strength of BSFRC.

Figure 16 presents the relationship between fiber hybrid content and the dynamic strength of BSFRC. It shows that the dynamic strength of concrete under different *w/c* ratios and impact pressures exhibits similar trends. Concrete reinforced with BF, SF, and their hybridization has higher impact strength than plain concrete but has a downward trend as the fiber hybrid content increases. It means that the two fibers can increase the impact resistance of concrete. As presented in Figure 9, fibers across the cracks can undertake parts of tension and play an inhibitory role in crack expansion. However, the probability of uneven dispersion and agglomeration of fibers increases by adding more BF and SF, leading to the decrease of impact strength of BSFRC. Whereas, SF is more effective than BF. Research shows that flexible fiber mainly improve the toughness of concrete while rigid fiber mainly improve the strength of concrete [43,44]. But the results in this study are different. This is because SF is a kind of macro synthetic fiber but has higher elastic modulus compared to the general flexible fiber. Thus, it can increase the dynamic strength, even better than BF. When the *w/c* is 0.68, concrete reinforced with hybrid fiber (BSC1) has lower impact strength than BC and SC. However, it is similar to SC but higher than BC as the *w/c* ratio decreases. It indicates that higher *w/c* ratio will weaken the effect of fiber hybridization.

**Figure 17:**
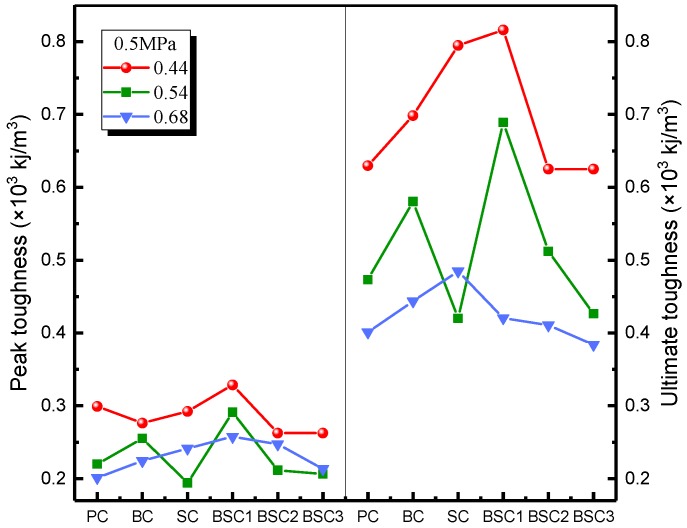
Fiber hybridization effect on impact toughness of BSFRC.

Figure 17 shows the relationship between impact toughness of concrete and fiber hybrid content of BSFRC under impact pressure of 0.5 MPa. Due to material inhomogeneities and experimental errors, the test results are a bit discrete, especially for the *w/c* ratio of 0.54. But in general, it can be seen that concrete reinforced with proper amounts of BF, SF, and their hybridization has higher impact toughness which means that the two fibers can increase the impact toughness of concrete but their hybridization has better toughening effect compared to SF and BF used individually. However, BSC1 has higher impact toughness compared to BSC2 and BSC3. It indicates that too much fiber will play a weaken role which is more obvious in lower *w/c* ratio. Besides, the impact toughness of concrete, especially the ultimate toughness, shows a great variation in lower *w/c* ratio but has only a little difference in higher *w/c* ratio. It indicates that the fiber hybridization effect is more obvious at lower *w/c* ratio.

## 4. Damage Dynamic Constitutive Model

### 4.1. Equation Establishment

The ZWT model [45], which was initially applied to polymer materials, is composed of a time-independent non-linear spring f(ε) and two time-dependent linear Maxwell bodies $\emptyset \left(\varepsilon ,\dot{\varepsilon }\right)$. The complete ZWT model is established as Equations (9)–(11). It can be seen that the model does not consider the influence of damage inside material. Differently from polymer materials, concrete materials in fact have many pores and microcracks inside. Besides, more cracks will be generated and propagate during the loading process. These defects will produce a depression effect on the mechanical performance and bring about complicated non-linear response of concrete. It is complicated to study the damage process from a micro perspective. But these defects play a role of weakening effect macroscopically. Wang et al. [46] considered that the damage evolution could be described by damage factor D, as given in Equation (12). In the SHPB test, two assumptions can be made to simplify the ZWT model. $\alpha \varepsilon^{2}$ and $\beta \alpha \varepsilon^{2}$ are too small to be taken into consideration and the stress and strain show linear relationship in the prophase stage. Therefore, the model can be established as Equation (13). The parameters of $E_{1}$ and $E_{2}$ can be obtained from the stress–strain curves of the static test and SHPB test. There are five parameters, namely, *m*, *a*, $E_{0}$, $\theta_{1}$ and $\theta_{2}$.

(9) $$\sigma =f\left(\varepsilon \right)+\emptyset \left(\varepsilon ,\dot{\varepsilon }\right)$$

(10) $$f\left(\varepsilon \right)=E_{0}\varepsilon +\alpha \varepsilon^{2}+\beta \alpha \varepsilon^{2}$$

(11) $$\emptyset \left(\varepsilon ,\dot{\varepsilon }\right)=E_{1}\int_{0}^{t}\dot{\varepsilon }\exp\left(-\frac{t-\tau }{\theta_{1}}\right)d\tau +E_{2}\int_{0}^{t}\dot{\varepsilon }\exp\left(-\frac{t-\tau }{\theta_{2}}\right)d\tau$$

(12) $$D=1-e^{-{\left(\frac{\varepsilon }{a}\right)}^{m}}$$

(13) $$\sigma =e^{-{\left(\frac{\varepsilon }{a}\right)}^{m}}\left[E_{0}\varepsilon +E_{1}\theta_{1}\dot{\varepsilon }\left(1-e^{-\;\frac{\varepsilon }{\dot{\varepsilon }\theta_{1}}}\right)+E_{2}\theta_{2}\dot{\varepsilon }\left(1-e^{-\;\frac{\varepsilon }{\dot{\varepsilon }\theta_{2}}}\right)\right]$$

where *m* and *a* are the scale and shape parameter of Weibull distribution. $E_{0}$ is the elastic modulus of the spring. $E_{1}$ and $\theta_{1}$ are elastic modulus and relaxation time of Maxwell I. $E_{2}$ and $\theta_{2}$ are elastic modulus and relaxation time of Maxwell II.

### 4.2. Analysis of Fitting

**Figure 18:**
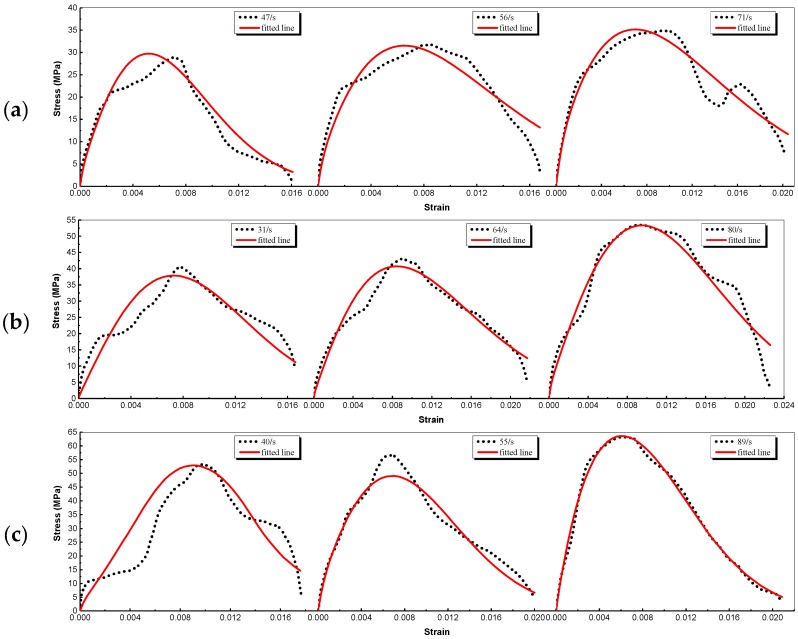
Experimental and fitted stress–strain curves at different strain rates: (**a**) BC–*w/c* ratio: 0.68; (**b**) BSC1–*w/c* ratio: 0.54; (**c**) SC–*w/c* ratio: 0.44.

Only part of the results is given because of paper length limitations. The fitted curves and parameters at various strain rates are plotted in Figure 18 and tabulated in Table 6, respectively. Similar regularity is derived from different types of BSFRC with different *w/c* ratios. It shows that the stress fitted by the proposed model agrees well with the test data. The level of conformity in the rising phase is very high but declines in the dropping phase. It can be explained as the stress in SHPB test is not uniform when the internal stress reaches its maximum value. Overall, Table 6 shows that as the strain rate increases, $E_{0}$, $E_{1}$ and $\theta_{1}$ experience very little change, $E_{2}$, $\theta_{1}$, $\theta_{2}$ and *m* increase while *a* decreases. It means that the dynamic elastic modulus of BSFRC is improved by strain rate. The increase of $\theta_{2}$ indicates that the strain rate response of BSFRC at high strain rate is much more clearly than that at low strain rate. Besides, Equation (12) shows that *m* has a greater effect upon D than *a*. D decreases with the increase of m and the decrease of *a*. The fitting result indicates that D is reduced which indicates that the microcracks propagation is restrained and the brittleness of BSFRC is improved with strain rate.

**Figure 19:**
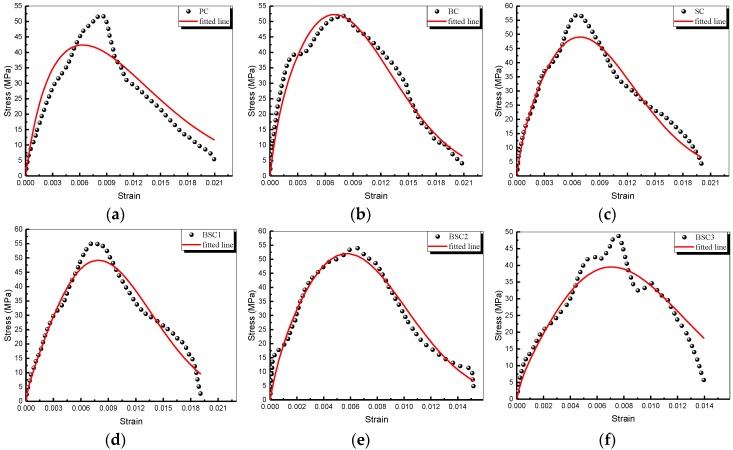
Experimental and fitted stress–strain curves with different *w/c* ratios: (**a**) PC; (**b**) BC; (**c**) SC; (**d**) BSC1; (**e**) BSC2; (**f**) BSC3.

The fitted curves and parameters with various hybrid ratios are plotted in in Figure 19 and tabulated in Table 7. It shows that the fitted stress agrees with the experimental results well. Table 7 shows that the regularities of the parameters are not clear. But we can know that the static and dynamic elastic constant of concrete are improved when added fibers. The relaxation time of BSFRC is reduced compared to plain concrete. Besides, it can be seen that m of BSFRC is increased which means that fiber can optimize the microstructure of concrete and decrease the damage inside. m of the BF and SF hybridization is higher than BC or SC. It indicates that the hybridization of BF and SF can restrain the propagation of microcracks and has a better optimization effect on the microstructure of concrete.

## 5. Microstructure Characteristics Analysis

### 5.1. Microscopic Appearance of Mortar

**Figure 20:**
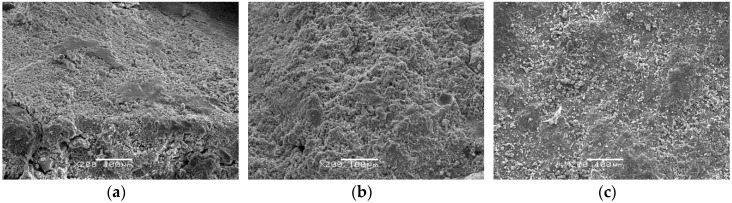
Microscopic appearance of mortar: (**a**) *w/c* ratio: 0.68; (**b**) *w/c* ratio: 0.54; (**c**) *w/c* ratio: 0.44.

The *w/c* ratio is an important factor affecting the failure mechanism of BSFRC. In this section, the microscopic appearance of mortar is studied with SEM equipment, as given in Figure 20. For higher *w/c* ratio (0.68), the hardened cement hydrates are loosely structured with many 1.5–3 μm microcracks and pores inside. As the *w/c* ratio declines, the content of cement increases gradually and the internal defects decrease. When *w/c* ratio is 0.44, there are few obvious cracks and large aperture pores and the aperture of the pores is reduced to 0.5–1 μm. The surface is gradually deposited with white Calcium Silicate Hydrate (C–S–H) gel hydrates. The mortar structure becomes stronger and more compact. Studies show that the hydration products of Portland cement are mainly composed of C–S–H gel phase, Solid hydrate (e.g., cement, calcium hydroxide $\mathrm{Ca}{\left(\mathrm{OH}\right)}_{2}$ and aluminate) and capillary pores [47]. C–S–H which affects the macro mechanical properties of concrete accounts for the largest proportion. However, when there is enough water, ettringite will be produced as well. The remarkable characteristic of ettringite is swelling which can increase the volume of the hydrated products [48], leading to many microcracks inside. As *w/c* ratio decreases, there is not enough water to generate ettringite and the C–S–H gel impel cement to form a more compact structure. With fewer microcracks and pores inside, the performance of concrete is clearly improved.

### 5.2. Interface Observation

**Figure 21:**
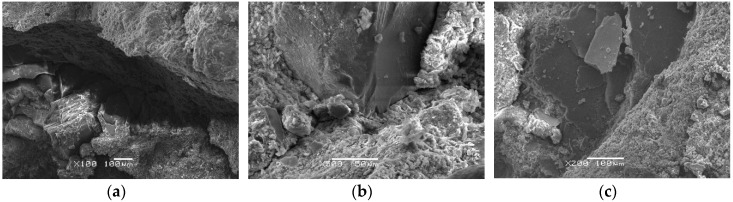
Interface between mortar and detritus: (**a**) *w/c* ratio: 0.68; (**b**) *w/c* ratio: 0.54; (**c**) *w/c* ratio: 0.44.

The cement hydration products and coarse aggregates are essentially two different materials and there exist interfaces between them. First, we study the interface characteristics between mortar and detritus of PC, as shown in Figure 21. It is observed that concrete with various *w/c* ratios has different degrees of damage in the ITZ. Concrete with the *w/c* ratio of 0.68 has apparent wide cracks and mortar is loosely bonded to aggregates. When the *w/c* ratio decreases, mortar of concrete with the *w/c* ratio of 0.54 has better encapsulation to aggregates but the mortar structure in the ITZ is relatively looser compared to that of concrete with the *w/c* ratio of 0.44. Besides, there is a significant difference between the microscopic appearance of ITZ and that of common mortar (CM) which is 500 μm away from aggregates. A large number of lamellar calcium hydroxide crystals of about 10 μm are produced in the ITZ with more defects and looser structure. Whereas, the mortar structure away from the aggregates is more compact.

**Figure 22:**
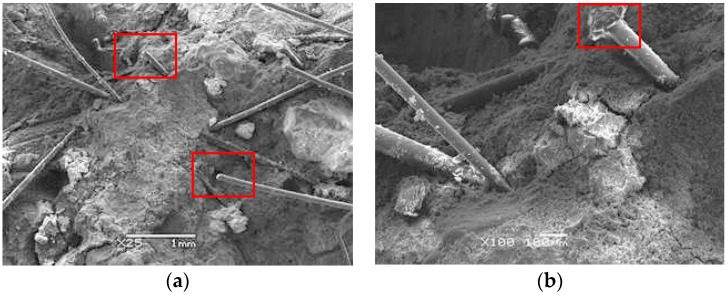
BF and SF distribution in concrete with *w/c* ratio of 0.54: (**a**) BF; (**b**) SF.

**Figure 23:**
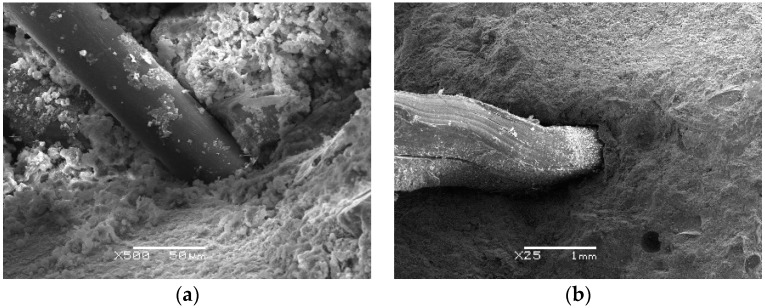
Interface between mortar and fibers: (**a**) Interface between mortar and BF of BC; (**b**) Interface between mortar and SF of SC.

Herein, we study the fiber distribution and interface between mortar and fibers. After observing several different samples, we found that they showed broadly similar distribution of BF and SF, as given in Figure 22. It shows that there are fibers parallel or inclined to the fracture surface which means that only part of the fibers plays an effective role. BF belongs to fine fibers and it is easy to bend and intertwine inside concrete. BF in fracture areas are mostly pulled apart, as shown in Figure 22a. SF is a kind of macro synthetic fiber with large diameter and seldom intertwines. SF in fracture areas are mostly pulled out, as shown in Figure 22b. Figure 23 shows the interface between mortar and fibers. Overall, the bonding interface between the two types of fibers and mortar is in great condition. The surface of BF is smooth and there is no obvious deformation which indicates that BF can improve the strength of concrete significantly. Instead, SF has serious plastic deformation after impact loading. It means that SF can absorb the external input energy effectively and improve the toughness of concrete significantly. Besides, there exists a thin layer of hydrate on the surface of BF. Although SF has poor water absorbability, the hydration products attached to SF increase the bonding strength between BF and mortar.

### 5.3. Energy Spectrum Analysis

**Figure 24:**
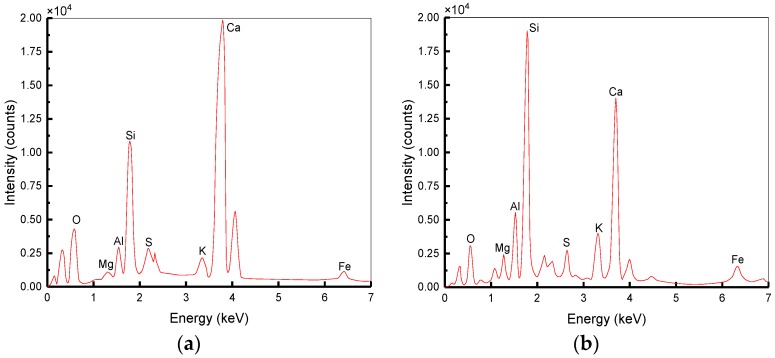
Energy spectrum in different areas of PC: (**a**) Spectrum1: Energy spectrum in the ITZ; (**b**) Spectrum2: Energy spectrum at 500 μm away.

The formation of ITZ is because of the gradient of *w/c* ratio caused by the penetration of water from mortar to aggregates surface and the thickness of ITZ is between 5 and 100 μm [49]. Three specimens were prepared for each condition and the average value were obtained. Figure 24 shows the difference in the energy spectrum of PC between ITZ and CM. The atomic percentage of different elements is tabulated in Table 8. it presents that the contents of both Ca and O are the highest in the ITZ and CM. However, the content of Si in the CM is higher than that in the ITZ. Ca/Si of ITZ is 4.25 and that of CM is 1.98. The results show that ITZ has less C–S–H but more $\mathrm{Ca}{\left(\mathrm{OH}\right)}_{2}$ than CM. It is the result of a higher *w/c* ratio near aggregates and $\mathrm{Ca}{\left(\mathrm{OH}\right)}_{2}$ exists in the form of crystallization with a looser structure and more pores. Instead, the *w/c* ratio of CM away from aggregates is lower and the content of C–S–H increases resulting in a compact structure. Therefore, the ITZ is an area with higher porosity and lower strength compared to CM.

**Figure 25:**
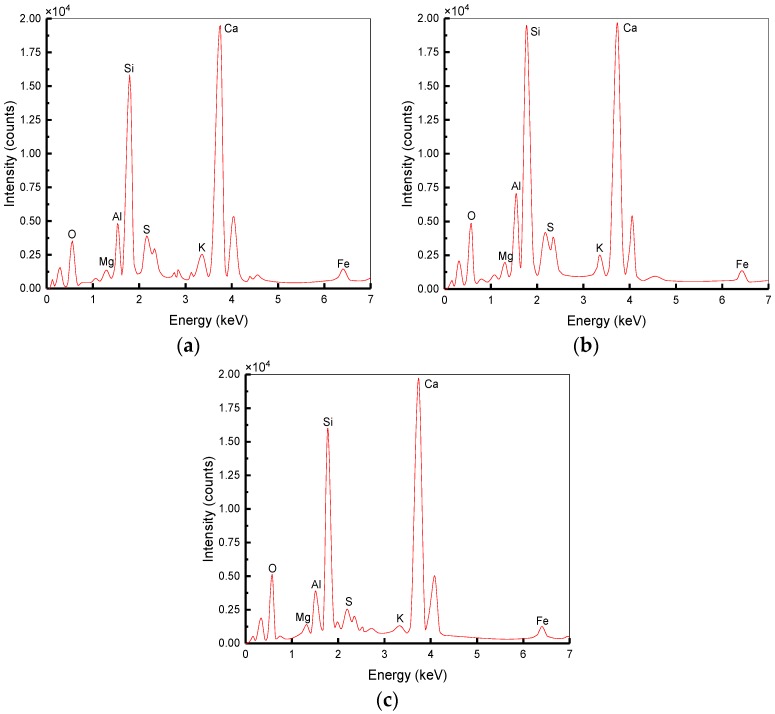
Energy spectrum in the ITZ of different concretes: (**a**) Spectrum3: Energy spectrum in the ITZ of BC; (**b**) Spectrum4: Energy spectrum in the ITZ of SC; (**c**) Spectrum5: Energy spectrum in the ITZ of BSC1.

Figure 25 shows the difference between energy spectrum of BC, SC and BSC1, and the atomic percentages of different elements are tabulated in Table 8. Ca/Si of BC, SC, and BSC1 are 2.75, 3.31, and 3.06, respectively, which are lower than that of PC (4.25). It indicates that BF, SF, and their hybridization can accelerate the hydration of cement in the ITZ of concrete and improve the strength of the ITZ area. Compared to SF, the lower Ca/Si of BC indicates that BF shows a better improvement effect, but both of them can clearly increase the strength of concrete.

## 6. Conclusions

The impact of fiber hybridization, strain rate, and *w/c* ratio upon the impact behavior of BSFRC was studied experimentally in this paper. The microstructure characteristics of BSFRC material was observed and analyzed as well. The main conclusions obtained are as follows:

- BF and SF are able to increase the static strength and their hybridization has a greater strengthening effect. The best hybrid ratio is 0.05% (BF)–0.25% (SF) for *w/c* ratios of 0.44 and 0.54, but higher fiber hybrid content is demanded for *w/c* ratio of 0.68.
- The impact test results indicate that BSFRC material is significantly affected by strain rate which can improve the dynamic mechanical parameters of BSFRC. The results show that DIF and $\mathrm{lg}\dot{\varepsilon }$ are linearly related. BSFRC with higher *w/c* ratio has a higher strain rate effect. As the strength of concrete decreases with increasing *w/c* ratio, the research indicates that the reduction of the concrete strength increases its strain rate sensitivity.
- Both fibers can improve the impact strength and toughness. Their hybridization has similar improvement effects on impact strength but can further increase the impact toughness and the best hybrid ratio is 0.05% (BF)–0.25% (SF). Higher *w/c* ratio will weaken the effect of fiber hybridization.
- The proposed model can well describe the impact behavior of BSFRC. The fitted parameters of $E_{2}$, $\theta_{1}$, $\theta_{2}$ and *m* are sensitive to strain rate. The static and dynamic elastic constant of BSFRC are improved compared to plain concrete. The BF and SF hybridization can restrain the damage evolution of concrete more obviously.
- The mortar structure gets more compact with more C–S–H and less $\mathrm{Ca}{\left(\mathrm{OH}\right)}_{2}$ with the decrease of *w/c* ratio. Besides, degree of damage in the ITZ of PC decreases as well but is worse than that in the CM. BF and SF with proper fiber content can be evenly distributed in concrete. BF are mostly pulled apart while SF are mostly pulled out in fracture areas. The energy spectrum results show that the hydration of cement in the ITZ is lower with more C–S–H and less $\mathrm{Ca}{\left(\mathrm{OH}\right)}_{2}$ than CM. However, the addition of BF and SF can promote the hydration of cement in the ITZ and improve the performance of ITZ.

In practical applications, concrete-like material has to inevitably suffer various impact loads, such as an earthquake, explosion, and wave or wind power. The hybridization of BF and SF is able to combine the advantages of both BF and SF. The impact resistance of concrete with a suitable amount of BF and SF added is improved remarkably. In addition, the production cost of BF and SF is lower than that of some other type of fibers, such as steel fiber and carbon fiber. Thus, BSFRC has great potential in concrete application.

## Figures and Tables

**Table 1:** Mixture proportion for concrete.

*w/c* Ratio	Proportion (kg/m^3^)			
	Water	Cement	Sand	Detritus
0.44	225	511	525	1120
0.54	225	417	589	1169
0.68	225	331	728	1116

**Table 2:** Physical and mechanical properties of BF and SF.

Fiber	Diameter (μm)	Fiber Length (mm)	Density (g/cm^3^)	Modulus of Elasticity (GPa)	Tensile Trength (GPa)	Elongation (%)
BF	15	12	2.65	93–110	4.5	3.15
SF	800	15	0.91	9	0.45	15

**Table 3:** Test conditions of basalt-macro synthetic polypropylene fiber reinforced concrete (BSFRC) specimens.

Group	BF	SF	*w/c*: 0.44		*w/c*: 0.54		*w/c*: 0.68	
	%	%	IP(MPa)	Label	IP(MPa)	Label	IP(MPa)	Label
PC	0	0	0	S-1	0	S-7	0	S-13
			0.3	1	0.3	19	0.3	37
			0.4	2	0.4	20	0.4	38
			0.5	3	0.5	21	0.5	39
BC	0.1	0	0	S-2	0	S-8	0	S-14
			0.3	4	0.3	22	0.3	40
			0.4	5	0.4	23	0.4	41
			0.5	6	0.5	24	0.5	42
SC	0	0.5	0	S-3	0	S-9	0	S-15
			0.3	7	0.3	25	0.3	43
			0.4	8	0.4	26	0.4	44
			0.5	9	0.5	27	0.5	45
BSC1	0.05	0.25	0	S-4	0	S-10	0	S-16
			0.3	10	0.3	28	0.3	46
			0.4	11	0.4	29	0.4	47
			0.5	12	0.5	30	0.5	48
BSC2	0.05	0.5	0	S-5	0	S-11	0	S-17
			0.3	13	0.3	31	0.3	49
			0.4	14	0.4	32	0.4	50
			0.5	15	0.5	33	0.5	51
BSC3	0.1	0.25	0	S-6	0	S-12	0	S-18
			0.3	16	0.3	34	0.3	52
			0.4	17	0.4	35	0.4	53
			0.5	18	0.5	36	0.5	54

IP means impact pressure; IP = 0 represents static tests.

**Table 4:** The dynamic characteristic parameters of BSFRC in the SHPB test.

Label	Strain Rate (/s)	Peak Stress (MPa)	Peak Strain	Ultimate Strain	Peak Toughness ×10^3^ kJ/m^3^	Ultimate Toughness ×10^3^ kJ/m^3^
1	37	45.71	0.00820	0.0218	0.1509	0.4112
2	64	52.00	0.00852	0.02104	0.2813	0.4965
3	82	57.36	0.00771	0.02201	0.2992	0.6296
4	35	46.54	0.00692	0.01922	0.2498	0.5616
5	58	51.80	0.00789	0.02099	0.3098	0.6268
6	85	60.25	0.00754	0.02224	0.2763	0.6983
7	40	53.04	0.00812	0.01770	0.2586	0.5240
8	55	56.83	0.00667	0.02021	0.2359	0.5576
9	89	62.99	0.00624	0.02086	0.2923	0.7947
10	33	52.12	0.00813	0.02014	0.2711	0.6157
11	61	55.17	0.00737	0.01906	0.2389	0.5760
12	90	65.10	0.00629	0.02124	0.3287	0.8159
13	38	47.83	0.00582	0.01958	0.2411	0.5826
14	60	54.09	0.00628	0.02044	0.2369	0.4834
15	79	59.57	0.00711	0.02138	0.2626	0.6249
16	32	48.32	0.00838	0.01856	0.2630	0.5238
17	54	48.94	0.00737	0.01709	0.2157	0.4678
18	84	55.86	0.00688	0.02128	0.2626	0.6249
19	28	34.12	0.00683	0.01722	0.1558	0.3736
20	55	38.06	0.00722	0.01663	0.1948	0.3283
21	75	46.27	0.00620	0.02201	0.2201	0.4732
22	34	34.41	0.00676	0.01764	0.1733	0.4482
23	58	38.72	0.00795	0.01868	0.2172	0.3898
24	77	47.57	0.00973	0.02196	0.2552	0.5805
25	35	40.80	0.00642	0.01432	0.1935	0.3922
26	63	44.23	0.00618	0.01782	0.2134	0.4820
27	72	51.89	0.00768	0.01985	0.1942	0.4201
28	31	40.99	0.00788	0.01606	0.1895	0.4205
29	64	43.36	0.00884	0.02179	0.2335	0.5006
30	80	53.48	0.00918	0.02256	0.2912	0.6891
31	37	36.54	0.00860	0.01823	0.2052	0.3826
32	60	36.78	0.00867	0.01757	0.1669	0.3927
33	83	45.87	0.00850	0.02232	0.2116	0.5121
34	40	36.82	0.00804	0.01798	0.1622	0.4052
35	57	36.35	0.00728	0.01975	0.1725	0.4525
36	91	47.20	0.00938	0.02196	0.2065	0.4265
37	41	27.37	0.00776	0.01120	0.1461	0.2340
38	62	29.75	0.00876	0.01637	0.1887	0.2271
39	85	32.61	0.01141	0.01918	0.2013	0.4012
40	47	29.09	0.00732	0.01604	0.1486	0.2225
41	56	31.94	0.00841	0.01677	0.1848	0.3768
42	71	34.87	0.00999	0.02044	0.2245	0.4437
43	39	30.48	0.00712	0.01594	0.1420	0.2094
44	56	33.56	0.00848	0.01692	0.2105	0.3898
45	73	36.98	0.01012	0.02053	0.2415	0.4847
46	40	28.78	0.00730	0.01578	0.1160	0.2271
47	48	31.29	0.00728	0.01370	0.1973	0.3648
48	77	35.27	0.00886	0.01705	0.2576	0.4204
49	42	28.80	0.00720	0.01321	0.1425	0.2599
50	54	30.23	0.00778	0.01824	0.1778	0.2585
51	76	31.81	0.01024	0.02136	0.2474	0.4107
52	37	29.04	0.00549	0.01196	0.1209	0.2506
53	52	30.51	0.00883	0.01462	0.2047	0.3021
54	79	32.88	0.00835	0.01819	0.2135	0.3840

**Table 5:** The linear fitting results of DIF function.

*w/c* Ratio	Group	Label	Functions	R	R^2^
0.44	BSC1	a4	$\mathrm{DIF}=0.59985\cdot \mathrm{lg}\dot{\varepsilon }+0.17387$	0.91064	0.82926
0.54	BSC1	b4	$\mathrm{DIF}=0.66064\cdot \mathrm{lg}\dot{\varepsilon }+0.08319$	0.80177	0.64283
0.68	PC	c1	$\mathrm{DIF}=0.68833\cdot \mathrm{lg}\dot{\varepsilon }+0.03273$	0.99152	0.98311
	BC	c2	$\mathrm{DIF}=1.27656\cdot \mathrm{lg}\dot{\varepsilon }-0.96958$	0.99690	0.99381
	SC	c3	$\mathrm{DIF}=0.87817\cdot \mathrm{lg}\dot{\varepsilon }-0.27113$	0.99293	0.98591
	BSC1	c4	$\mathrm{DIF}=0.89602\cdot \mathrm{lg}\dot{\varepsilon }-0.25745$	0.99275	0.98555
	BSC2	c5	$\mathrm{DIF}=0.45952\cdot \mathrm{lg}\dot{\varepsilon }+0.39475$	0.99826	0.99652
	BSC3	c6	$\mathrm{DIF}=0.45560\cdot \mathrm{lg}\dot{\varepsilon }+0.41188$	0.99717	0.99434

**Table 6:** The fitted parameters of BSFRC under different strain rate.

*w/c* Ratio	Group	Strain Rate (/s)	$E_{0}\;(\mathrm{GPa})$	$E_{1}\;(\mathrm{GPa})$	$E_{2}\;(\mathrm{GPa})$	$\theta_{1}\;(s)$	$\theta_{2}\;(\times 10^{-6}\;s)$	*m*	*a* (×10^−3^)
0.44	SC	40	0.538	8.7	10.4	1.37	5.992	2.154	14.23
	SC	55	1.512	8.7	14.3	2.15	11.56	1.982	10.22
	SC	89	0.376	8.7	18.4	2.37	2.225	1.933	10.53
0.54	BSC1	31	0.694	7.9	13.4	1.35	1.181	1.954	10.28
	BSC1	64	1.013	7.9	14.8	1.56	1.225	1.865	11.36
	BSC1	80	1.410	7.9	15.9	1.97	2.348	1.800	13.40
0.68	BC	47	1.265	8.1	11.8	1.18	5.188	1.730	7.36
	BC	56	0.793	8.1	12.8	1.26	6.44	1.350	8.62
	BC	71	0.792	8.1	14.4	1.32	7.244	1.310	9.36

**Table 7:** The fitted parameters of BSFRC with different fiber hybrid ratios.

*w/c* Ratio	Group	Strain Rate (/s)	$E_{0}\;(\mathrm{GPa})$	$E_{1}\;(\mathrm{GPa})$	$E_{2}\;(\mathrm{GPa})$	$\theta_{1}\;(s)$	$\theta_{2}\;(\times 10^{-6}\;s)$	*m*	*a* (×10^−3^)
0.44	PC	64	1.545	7.5	11.8	4.84	23.96	1.350	9.58
	BC	58	0.765	8.5	16.9	1.92	15.34	1.928	10.92
	SC	55	1.512	8.7	14.3	2.15	11.56	1.852	10.22
	BSC1	61	1.480	7.9	12.5	4.32	4.163	2.129	11.48
	BSC2	60	2.790	8.8	15.3	2.68	11.78	2.186	8.77
	BSC3	54	0.787	7.4	11.5	3.22	6.297	2.103	10.37

**Table 8:** The atomic percentage of different energy spectrums.

Element	Atomic Percentage (%)							
	O	Mg	Al	Si	S	K	Ca	Fe
Spectrum 1	45.88	0.65	2.49	8.96	1.39	1.19	38.04	1.40
Spectrum 2	34.13	2.79	6.67	15.51	1.64	4.63	30.69	3.94
Spectrum 3	36.44	0.66	4.29	14.21	2.12	1.52	39.13	1.63
Spectrum 4	38.84	1.29	5.25	11.52	2.21	0.95	38.11	1.83
Spectrum 5	45.56	0.98	2.92	11.70	0.98	0.37	35.86	1.63
